# A systematic review of studies measuring health-related quality of life of general injury populations

**DOI:** 10.1186/1471-2458-10-783

**Published:** 2010-12-23

**Authors:** Suzanne Polinder, Juanita A Haagsma, Eefje Belt, Ronan A Lyons, Vicki Erasmus, Johan Lund, Ed F van Beeck

**Affiliations:** 1 Erasmus Medical Centre, Department of Public Health, Rotterdam, the Netherlands; 2 College of Medicine, Swansea University, Swansea, UK; 3 Faculty of Medicine, University of Oslo, Oslo, Norway

## Abstract

**Background:**

It is important to obtain greater insight into health-related quality of life (HRQL) of injury patients in order to document people's pathways to recovery and to quantify the impact of injury on population health over time. We performed a systematic review of studies measuring HRQL in general injury populations with a generic health state measure to summarize existing knowledge.

**Methods:**

Injury studies (1995-2009) were identified with main inclusion criteria being the use of a generic health status measure and *not *being restricted to one specific type of injury. Articles were collated by study design, HRQL instrument used, timing of assessment(s), predictive variables and ability to detect change over time.

**Results:**

Forty one studies met inclusion criteria, using 24 different generic HRQL and functional status measures (most used were SF-36, FIM, GOS, EQ-5D). The majority of the studies used a longitudinal design, but with different lengths and timings of follow-up (mostly 6, 12, and 24 months). Different generic health measures were able to discriminate between the health status of subgroups and picked up changes in health status between discharge and 12 month follow-up. Most studies reported high prevalences of health problems within the first year after injury. The twelve studies that reported HRQL utility scores showed considerable but incomplete recovery in the first year after discharge.

**Conclusion:**

This systematic review demonstrates large variation in use of HRQL instruments, study populations, and assessment time points used in studies measuring HRQL of general injury populations. This variability impedes comparison of HRQL summary scores between studies and prevented formal meta-analyses aiming to quantify and improve precision of the impact of injury on population health over time.

## Background

Worldwide, injuries are recognized as a major concern in public health, being the predominant cause of deaths in adults aged 1- 45 years, and an important cause of disabilities [1,2]. The number of survivors of severe injuries has rapidly grown due to substantial improvements in trauma care. This has resulted in a shift of focus from mortality towards disability of injury patients. Disability (i.e. reduced levels of functioning resulting from diseases or injuries [3]) is increasingly seen as an important component of a population's health and for the field of injury prevention and trauma care [4].

Disability is a complex construct and can be measured using functional instruments or generic or disease specific HRQL measures, where disability represents the gap between measured and perfect HRQL. To enable straightforward comparisons with other disease groups and with general population norms, it is necessary to measure the consequences of injuries using generic health status measures (for instance the SF-36 or the EQ-5D). Some HRQL instruments generate a summary score (utility) that can contribute to a composite health outcome measure [1]. It has become common practice to quantify the impact of diseases and injuries on population health with the help of composite health outcome measures, such as quality-adjusted life years (QALYs) and disability-adjusted life years (DALYs) [4,5]. Sound epidemiological data on the incidence, severity and duration of the functional consequences of injuries are needed to make valid estimates of the years lived with disability due to injuries in the population. Data on all dimensions of functioning relevant to injuries are needed to describe the pattern of recovery or residual disability of injury patients over time. With the help of these data, the impact of injury on population health over time can be quantified. Measuring the impact of injury is particularly challenging due to the large variation in injury types and severity. The European Consumer Safety Association has published guidelines for the conduction of follow-up studies measuring injury-related disability based on a narrative literature search of papers from1995-2005 [1]. They concluded that in the injury field there is lack of consensus on preferred HRQL instruments and study designs [1]. However, this review only included 14 studies that measured HRQL in general injury populations. Derrett et al conducted a more recent systematic literature search of injury specific and generic studies measuring outcome after injury but restricted this to studies using the EQ-5D outcome measure. They called for further comprehensive population-level research exploring outcomes after injury, and particularly for studies focusing on 'all injury' [6]. It is clear that there is a need to obtain greater insight into patterns of HRQL in comprehensive injury populations in order to document people's pathways to recovery and to quantify the impact of injury on population health over time [1,6,7]. Given the appearance of additional studies after 2005, and the variety of generic measures in this field of research, the current systematic review was conducted to describe the up to date state of knowledge in this field and hopefully contribute to further consensus development on preferred methodologies within the injury research field.

This review focused on the measurement of HRQL with a generic instrument among general injury populations. The following key questions were addressed: a) which generic instruments were used?, b) how were these instruments administered?, c) at which time points was HRQL assessed?, and d) did the instrument measure changes over time and predictors for HRQL? Furthermore, in anticipation of substantial heterogeneity preventing formal meta-analysis we aimed to produce a narrative summary of study outcomes to improve insight into general recovery patterns and residual disability.

## Methods

### Data sources and search strategy

We conducted a literature search aiming to identify empirical studies on injury-related disability. Searches of eligible studies were conducted in PubMed (Medline), Web of Science, Embase, and PsychInfo. All peer-reviewed articles published in the period January 1995 to 2009 were included in the searches. An electronic search strategy was developed in collaboration with a librarian with extensive experience in systematic reviews. Search terms used were: 'wounds and injuries', 'health status indicators', 'disability evaluation', 'functional outcome', 'health status measure', and 'cohort studies' (details in Additional file 1). Keywords were matched to database specific indexing terms. In addition to database searches, reference lists of review studies and articles included in the review were screened for titles that included key terms. More detailed information on the review can be found in the report compiled for the INTEGRIS (Integration of European Injury Statistics) project [8].

### Selection criteria

The inclusion criteria were studies using HRQL instruments in injury patients irrespective of the underlying injury, published in English or German in a peer-reviewed journal in the period 1995-2009. We focused on 'all injury' studies and therefore excluded injury-specific studies (for instance limited to brain injuries or hip fractures). Studies concerning people other than the injury victim were excluded, e.g. studies of impact of witnessing trauma. We included studies that reflected the definition of injuries used by the World Health Organization (WHO) as 'relatively sudden discernible effects due to body tissue damage from energy exchanges or ingestion of toxic substances but not due to medical adverse events, and obtained from health care settings' [9]. We included only longitudinal studies in line with the EuroSafe guidance [1].

### Data extraction

Relevant papers were selected by screening the titles (first step), abstracts (second step) and entire articles (third step), retrieved through the database searches. During each step the title, abstract or entire article was screened to ensure that it met the selection criteria listed above. This screening was conducted independently by two researchers (SP and EB). Two experts in this field (RL and JL) checked a sample of the abstracts (n = 50) on the inclusion criteria, to quality assure the process. Full articles were critically appraised by two reviewers (EB and SP), using data extraction forms developed for this study in a Microsoft Access-database.

Data were tabulated from studies that used a HRQL instrument and that reported a utility score or summary score, to give greater insight into the recovery patterns and changes over time of the different instruments. Utility scores are based on preferences or values related to health states and are derived from approaches used in decision theory and economics. Utility scores represent the total HRQL status of a person in a number on a 0 (or <0)-1 scale (where 0 indicates death or maximum amount of disability, and 1 being optimal health status). The EQ-5D and the SF-6D are examples of HRQL assessment instruments that produce such utility scores. Some instruments report a disability score (in which 1 represents the maximum amount of disability) for instance the WHODAS II [10]. Although there are some differences between the concepts, utility and disability scores will be referred to as summary scores in the remainder of this paper.

## Results

### Literature search

The database search identified 6291 titles of potentially relevant articles. In the first round (scanning the titles) 6031 articles were excluded. The main reasons for exclusion were studies which did not concern injury or were restricted to specific injuries. Of the remaining 260 articles, 165 were excluded after scanning the abstracts, mainly because the paper did not include self-reported HRQL measures. This resulted in scanning 95 full texts of which 54 did not meet our inclusion criteria, leading to inclusion of 41 articles. In this last round the main reason for excluding a full-text article was not using a generic health status measure or not describing the general injured population in sufficient detail (Additional file 2).

### Study characteristics

Of the 41 studies included in our systematic review, most were conducted in the US (n = 12, [11-22]), followed by the UK (n = 7, [23-29]), Australia (n = 5, [30-33]), and the Netherlands (n = 5, [34-38]) (Table 1 and 2). Sample sizes of the studies varied widely, between 35 and 13,649 participants, with most studies having sample sizes between 100 and 300 participants (n = 17, Table 1 and 2). Nine studies measured HRQL after injury for children and adolescents (shaded articles in table 1 and 2). All studies included non selected cases from 'all injury' populations and included injuries of different levels of severity.

**Table 1:** Study characteristics and methodological aspects of follow-up studies measuring health-related quality of life of injury patients (in order of nr of HRQL instruments used - bold author names are studies of children)

Author, year, country	Study population	HRQL instrument (mode of administration)	Follow up (time points and response rates)	Changes over time	Predictors for HRQL
Meerding, 2004, Netherlands [35]	ED and/or admitted Age 15+ (n = 4639)	EQ-5D (Self-completed)	2 months (39%) 5 months (24%) 9 months (12%)	Improvements between 2 and 5 months, no further improvement between 5 and 9 months	HRQL associated with body region injured

**Polinder, 2005, Netherlands **[36]	ED and/or admitted Age 5-14 (n = 1221)	EQ-5D (Self-completed, age < 13 proxy)	2.5 month (43%) 5 months (31%) 6 months (30%)	Improvements between 2.5 and 5 months, and between 5 and 9 months	Hospital admission and female gender were predictive for long-term HRQL

Polinder, 2007, Netherlands [37]	ED and/or admitted Age: >14 (n = 8564)	EQ-5D (Self-completed)	2.5 month (37%) 5 months (28%) 9 months (27%) 24 months (21%)	Improvement among non admitted patients until 5 months, and among admitted patients until 24 months	Hospitalization, age and sex (females), type of injury and comorbidity were significant predictors of poor functioning in the long term

Vasquez, 1996, Spain [56]	Admitted ICU patients (n = 351)	GOS (Self-completed)	Admission 1 year 2 year (% not available)	Quality of life improved the first year and between 1 and 2 years, but after 2 years still below pre-admission summary scores	Long-term HRQL was associated with age, injury severity, and previous quality of life

Hetherington, 1995, UK [28]	Trauma helicopter patients (n = 100)	FIM (Face to face interviews)	Acute 3 months 6 months (93%)	Improvements in mobility and self care between 3 and 6 months	HRQL associated with length of hospital stay

**Gofin, 1997, Israel **[57]	Age 4-17 (n = 281)	ICIDH (Telephone parent interviews)	Immediately 6 months (85%)	Improvements until 6 months after injury	HRQL is positively associated with ISS

**Gofin, 1995, Israel **[58]	Age 0-17 (n = 432)	ICIDH (Telephone parent interviews)	Immediately 6 months (85%)	Improvements until 6 months after injury	HRQL associated with child's age and parental proxy age

Holbrook, 1998, US [14]	>24 hours admitted in trauma center Age 18+ GCS >11 (n = 1048)	QWB-scale (Face to face interviews)	Pre-injury Discharge 6 months (79%)	Significant degree of functional limitations at discharge compared to pre-injury scores.	Post-injury depression, PTSD, serious extremity injury, and length of stay were significant predictors of HRQL

Holbrook, 1999, US [15]	>24 hours admitted in trauma center Age 18+ GCS >11 (n = 1048)	QWB-scale (Face to face interviews)	Pre-injury Discharge (79%) 12 months (79%) 18 months (74%)	Improvement between discharge and 12 months, but no improvements between 12 and 18 months.	Post-injury depression, PTSD, serious extremity injury, and intensive care unit days were significant independent predictors of HRQL

Holbrook, 2004, US [16]	>24 hours admitted in trauma center Age 18+ GCS >11 (n = 1048)	QWB-scale (Face to face interviews)	Discharge (79%) 6 months (79%) 12 months (74%) 18 months (74%)	Improvement between 6 and 12 months	Gender (women) was a significant independent predictors of HRQL at all follow-up time points

Gabbe, 2007, Australia [31]	Admitted Age 18 -64 (n = 1033)	SF-12 (Telephone interviews)	Pre-injury 12 months (69%)	After 12 months patients were not returned to their pre-injury status	Compensable patients were more likely than non-compensable patients to report moderate to severe HRQL

Brenneman, 1997, Canada [59]	Admitted ISS >10 (N = 195)	SF-36 (Telephone interviews)	Discharge (56%) 12 months (44%)	Improvements between discharge and 12 months	Better scores on 7 dimensions of the SF-36 for patients who returned to work

Michaels, 1999, US [20]	Admitted to trauma centre Age 18+ (n = 247)	SF-36 (Self-completed)	Admission (100%) 6 months (75%) 12 months (51%)	Improvements between baseline and 6 months, and between 6 and 12 months	Mental health (PTSD) is an independent predictor of HRQL

Kopjar, 1996, Norway [60]	Treated for injury Age 16-78 (n = 775)	SF-36 (Self-completed)	6-10 weeks (61%) 24-28 weeks (63%)	Improvements between 2 and six months	HRQL associated with activity restrictions

**Macpherson, 2003, Canada **[39]	Hospital inpatients Age 2-15 ISS >12 (n = 489)	WeeFIM (Telephone interviews)	Discharge 6 months (73%)	Improvement between discharge and 6 months	Injury mechanism is an independent predictor of HRQL

**Aitken, 2002, US **[11]	Admitted to children's hospital Age 3-18 ISS > 3 (n = 310)	CHQ PF-50, WeeFIM (Parent interview, child Self-completed)	Discharge 63%) 1 month (56%) 6 months (45%)	Improvements between 1 and 6 months	HRQL associated with level of injury severity

**Winthrop, 2005, US **[21]	Admitted Age 1-18 ISS > 8 (n = 180)	CHQ, FIM (Face-to-face interviews)	Discharge (90%) 1month 6 months (87%) 12 months	Improvements between baseline and 1 month, and between 1 and 6 months	HRQL associated with injury severity

Baldry Currens, 2000, UK [24,25]	Survivors of major trauma Admitted >3 days Age 5+ (n = 251)	FIM, GOS (Telephone interviews)	3 months (80%) 6 months 12 months > 24 months	Improvements between 3 and 6 months No further improvement between 6 months and 1 year	HRQL associated with major vs. minor injury and body region injured

Gillen, 2004, US [13]	Age 20+ (n = 114)	SF-36, HAQ (Telephone interviews)	1 week 2 weeks 1 month 3 months (79%)	Improvements between 1 week and 2 weeks, between 2 weeks and 1 month, and between 1 and 3 months.	The SF-36 and the HAQ were responsive to clinical changes in varying conditions and the SF-36 was sensitive to changes in traumatic injuries.

Kiely, 2006, US [18]	Age 18-55 ISS > 8 and all patients with age 55+ (n = 312)	SF-36, FIM (Telephone interview or self-completed)	1 month (63%) 6 months (39%)	Improvements between 1 and 6 months post injury	Functional status, PTSD, social support, and depression were predictors of HRQL

Soberg, 2007, Norway [43]	Admitted to trauma centre ISS > 15 Age 18-67 (n = 169)	SF-36, WHODASH II (Self-completed)	6 weeks (62%) 1 year (61%) 2 years (60%)	Improvements, except for mental and general health between 6 wk and1 year. Between 1 and 2 years almost no improvements.	Profession, injury severity, pain, and physical, cognitive, and social functioning made independent contributions to WHODAS II 2 years after injury

Evanoff, 2002, US [12]	Workers > 5 days workdays lost (n = 934)	SF-36, SF-12, DASH short version (Face to face interviews)	Baseline (33%) 6 months (27%)	Improvement over 6 months, after 6 months continuing HRQL	No

Watson, 2005, Australia [33]	Admitted Age 18-74 (n = 221)	SF-36, AQol, SF-6D (Face-to-face interviews)	Pre-injury 1, 6, 12 weeks 6 months 12 months (84%)	Significant improvement of functional outcome till 6 months; no significant difference in summary scores at 6 and 12 months post-injury	The AQoL showed good discrimination between groups for type of injury, body region injured and severity of injury

Watson, 2007, Australia [33]	Admitted Age 18-74 (n = 186)	SF-36, AQol, SF-6D (Face-to-face interviews)	Pre-injury 12 months (88%)	No difference between summary scores at baseline and 12 months after injury for patients that completely recovered	Gender, age, working status were predictors for complete recovery after one year

Gabbe, 2008, Australia [32]	Age 15-80 ISS > 15 (n = 243)	FIM, Modified FIM, GOS, GOS-E (Telephone interviews with participants or care provider)	Discharge 6 months (97%)	Improvement between discharge and 6 months, except for the cognition component of the FIM	HRQL associated with discharge destination and head injury vs. no head injury

Sutherland, 2005, UK [29]	Admitted Age 16-70 (n = 200)	SF-36, SF-6D, MFA, GHQ (Self-completed)	2 months (79%) 6 months (75%)	No improvement between 2 and 6 months	No

**AMA-guides **= American Medical Association guides; **BDS **= Bull Disability Scale; **CFS **= Cognitive Function Scale; **CHQ PF-50 **= Parent Completed version of the CHQ; **CHQ **= Child Health Questionnaire; **EQ5 D **= European Quality of life instrument-5 dimensions;; **FIM **= Functional Independence Measure; **GHQ **= General Health Questionnaire; **GOS **= Glasgow Outcome Scale; **HAQ **= Health Assessment Questionnaire; **HOBQ **= Health Outcomes Burn Questionnaire for Children; **ICIDH2 **= 25 item scale for measuring functional outcome by the International Classification of Impairments Disabilities and Handicaps; **MFA **= Musculoskeletal Functional Assessment; **NHP **= Nottingham Health Profile; **OPCS **= Office of Population Census and Surveys national survey of disability in Great Britain; **QOL **= Satisfaction with Quality of Life instrument; **QWB **= Quality of Well Being scale; **RDS **= Rosser Disease Score; **RTW **= Return To pre-injury Work status; **SF-6D **= Medical Outcome Study Short Form-6 dimensions; **SF-12 **= Medical Outcome Study Short Form-12 items; **SF-36 **= Medical Outcome Study Short form-36 items; **SIP **= Sickness Impact Profile; **TOP **= Trauma Outcome Profile; **WeeFIM **= Pediatric version of the FIM; **WODASII **= World Health Organization Disability Assessment Schedule version II; **WHOQOL-BREF **= short version of the World Health Organization Quality of life.

**Table 2:** Study characteristics and methodological aspects of studies measuring health-related quality of life of injury patients at one time point (in order of nr of HRQL instruments used - bold author names are studies of children)

Author, year, country	Study population	HRQL instrument (mode of administration)	Follow up (response rates)	Predictors for HRQL
Braithwaite, 1998, UK [26]	Severe injuries ISS > 15 Age 15+ (n = 212)	BDS (Face to face interview)	5 years (75%)	HRQL associated with body region injured

Korosec, 2006, Slovenia [61]	ICU patients (n = 98)	EQ5D (Telephone interview)	2 years (% not available)	No

**Aitken, 1999, US and Canada **[22]	Hospitalized Age 7-18 (n = 13649)	FIM (Filled out by doctors)	Discharge (80%)	Functional outcome associated with type of injury. Lower extremity fractures caused more limitations at discharge as compared to other injuries

**Evans, 2003, UK **[27]	Age 11-24 ISS > 15 (n = 125)	OPCS (Face to face interview)	5 years (87%)	Not measured

Holtslag, 2007, Netherlands [34]	Age 16+ ISS > 15 (n = 359)	SIP (Self-completed)	Between 12-18 months (93%)	Age, comorbidity, and type of injury were predictors of HRQL

MacKenzie, 2002, US [19]	Admitted > 72 hours or to ICU Aga 18-59 years (n = 1587)	SF-36 + cognitive functioning scale (Face to face interview)	12 months (78%)	Cognitive functioning and head injury were predictors of HRQL

Alves, 2009, Brazil [62]	ED and admitted >24 hours Age 16-65 GCS > 9 and ISS > 5 (n = 35)	WHOQOL-BREF (face-to-face interview)	6 months (88%)	Hospitalization, age, and sex were predictors for functional impairment in the physical domain

Airey, 2001, UK [23]	Admitted survivors of major trauma ISS > 15 (n = 367)	SF-36, OPCS (Face to face interviews)	5 years (84%)	HRQL associated with injury severity and general health perception

Pirente, 2001, Germany [63]	Admitted and 'severely injured' (n = 56)	SF-36, TOP (Telephone interview or self-completed)	12 months (77%)	HRQL among trauma patients higher than control group on al SF-36 dimensions (no injury)

Holtslag, 2008, Netherlands [64]	Age 16+ ISS > 15 (n = 359)	GOS, EQ-5D (self-completed)	Between 12-18 months (93%)	Injury type and comorbidity were significantly associated with HRQL

**Janssens, 2009, Netherlands **[38]	Admitted to trauma center Ag < 16 ISS > 15 (n = 40)	GOS and GOSE, AMA guides (Self-completed and face-to-face interview)	7 years (70%)	Good discrimination could be made between respondents with different levels of functional impairment

Dimopoulou, 2004, Greece [65]	Admitted multiple trauma patients (n = 117)	GOS, NHP, RDS (Telephone interview)	12 months (74%)	HRQL associated with injury severity ISS and severe head trauma were independent predictors of poor HRQL

Keyes, 2001, US [17]	Workers with > 3 days work loss (n = 402)	SF-36, HAQ, QOL (Telephone interview or self-completed)	2 years (93%)	No

Stalp, 2001, Germany [66]	Admitted ISS > 15 (n = 150)	SF-12, FIM, GOS, MFA (Self-completed)	24 months (81%)	HRQL associated with body region injured

Stalp, 2002, Germany [67]	Admitted ISS > 15 (n = 312)	SF-12, FIM, GOS, MFA (Self-completed)	24 months (81%)	HRQL associated with body region injured (highest for lower extremity injury)

**AMA-guides **= American Medical Association guides; **BDS **= Bull Disability Scale; **CFS **= Cognitive Function Scale; **CHQ PF-50 **= Parent Completed version of the CHQ; **CHQ **= Child Health Questionnaire; **EQ5 D **= European Quality of life instrument-5 dimensions;; **FIM **= Functional Independence Measure; **GHQ **= General Health Questionnaire; **GOS **= Glasgow Outcome Scale; **HAQ **= Health Assessment Questionnaire; **HOBQ **= Health Outcomes Burn Questionnaire for Children; **ICIDH2 **= 25 item scale for measuring functional outcome by the International Classification of Impairments Disabilities and Handicaps; **MFA **= Musculoskeletal Functional Assessment; **NHP **= Nottingham Health Profile; **OPCS **= Office of Population Census and Surveys national survey of disability in Great Britain; **QOL **= Satisfaction with Quality of Life instrument; **QWB **= Quality of Well Being scale; **RDS **= Rosser Disease Score; **RTW **= Return To pre-injury Work status; **SF-6D **= Medical Outcome Study Short Form-6 dimensions; **SF-12 **= Medical Outcome Study Short Form-12 items; **SF-36 **= Medical Outcome Study Short form-36 items; **SIP **= Sickness Impact Profile; **TOP **= Trauma Outcome Profile; **WeeFIM **= Pediatric version of the FIM; **WODASII **= World Health Organization Disability Assessment Schedule version II; **WHOQOL-BREF **= short version of the World Health Organization Quality of life.

Studies of disability restricted to the most severely injured patients are increasingly conducted. These studies used different severity scales and cutoff points for decision criteria for 'major trauma patients'. There were 10 studies that clearly described inclusion criteria using the most widely used inclusion definition of major trauma patients, namely an Injury Severity Score (ISS) > 15. Threshold scores from the Abbreviated Injury Scale (AIS), Glasgow Coma Score (GCS) or admission to a 'trauma center' for longer than 24 hours were also used as inclusion criteria.

Particularly for studies of low to moderate severity injury populations, e.g. emergency department (ED) attendees, there were difficulties in acquiring acceptable response and retention rates. Higher rates were more often reported in studies where outcome measures were administered by clinicians.

### Measurement of health related quality of life

#### Study design

**Figure 1:**
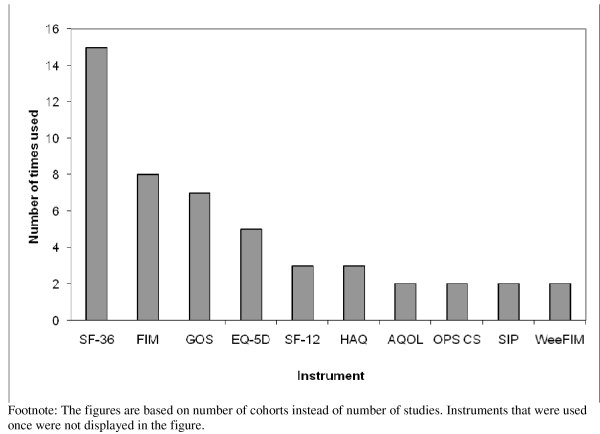
**Instruments used in patient follow-up studies for assessing injury-related disability**. SF-36 = Medical Outcome Study Short form-36 items; FIM = Functional Independence Measure; GOS = Glasgow Outcome Scale; EQ5 D = European Quality of life instrument-5 dimensions; SF-12 = Medical Outcome Study Short Form-12 items; SF-6D = Medical Outcome Study Short Form-6 dimensions; QWB = Quality of Well Being scale; HAQ = Health Assessment Questionnaire; AQoL = Assessment of Quality of Life instrument; OPCS = Office of Population Census and Surveys national survey of disability in Great Britain; ICIDH = 25 item scale for measuring functional outcome by the International Classification of Impairments Disabilities and Handicaps; CHQ = Child Health Questionnaire.

Twenty-four different instruments were used to assess HRQL or functional status. Of the available generic instruments, the SF-36 (n = 15), (Wee)FIM (n = 10), GOS (n = 7) and the EQ-5D (n = 5) have most often been applied among injury patients (see Figure 1). Half of the studies used more than one instrument to measure HRQL (two instruments: n = 10; and more than two instruments: n = 10). None of these studies used an injury specific measure besides a generic measure. In the nine studies among children, only three used a children's instrument [21,39,40]. All three studies also included an 'all-ages' instrument.

**Figure 2:**
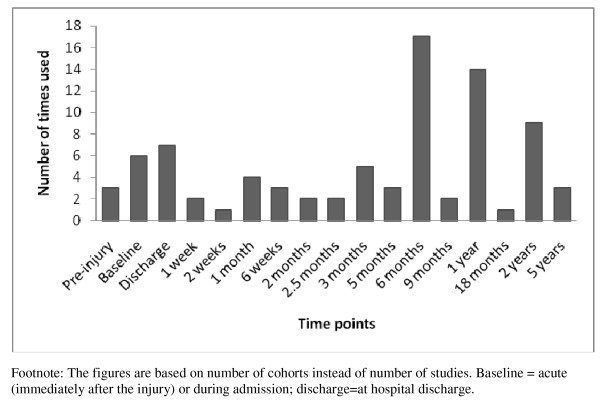
**Time points at which injury-related disability was assessed**.

Twenty-six studies used a longitudinal design with multiple assessments over time. HRQL was assessed most frequently at discharge, six months, one year, and two years following injury (Figure 2). There were five papers that assessed pre-injury health status, i.e. after the injury patients experienced the shock of sustaining an injury [14,15,33,41]. Variation was also apparent in the mode of administration with a mixture of self-completed questionnaires (n = 14), face to face interviews (n = 13), and telephone interviews (n = 14) of which 4 were telephone proxy interviews with parents, being used.

#### Predictors for HRQL

High prevalence's of health problems within the first year after injury were a common finding of the studies. Studies often included a large variety of associated variables which affected disability scores. Predictive variables frequently reported included injury severity, type of injury, gender, mental health status and comorbidity. The generic instruments showed similar differences between subgroups. The SF-36 and the EQ-5D were reported to be able to discriminate between the health status of injured patients and non-injured persons and between patients with different types of injuries (e.g. [13,33,42,43]). Among the majority of studies hospitalization, injury type and/or mechanism, and injury severity were predictive for long-term disability.

#### Changes over time

All HRQL instruments demonstrated improvements in health over time within the first 3 to 6 months after the injury. Most studies reported improvement in HRQL between discharge and one year after injury, and studies of 'severe' injuries also found improvements one to two years following the injury (Table 1).

**Figure 3:**
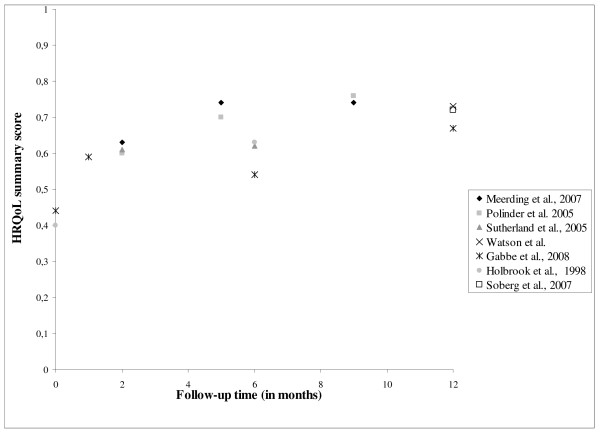
**Reported HRQL summary scores and utility scores over time (≤12 months) of general injury populations**.

There were twelve studies that reported HRQL utility scores. Table 3 shows that the injury populations differed considerably. All studies reported measurable recovery in the first year after injury. Among the severely injury patients there was some evidence of further improvement in the second year. Figure 3 provides an overview of the studies that reported HRQL summary scores over time in the 12 months following discharge among patients aged 15 or older. Overall, HRQL improves in the first year after discharge, although the large variation in HRQL instruments, study population and time points at which HRQL was assessed impedes comparison of HRQL summary scores between studies.

**Table 3:** Reported utility scores and summarized study descriptions

Study	Instrument	Sample size (n)	time point	Index mean (SD)	Study population
**ALL INJURY PATIENTS**					

Keyes, US [19]	QOL	854	24 months	0.70	Adults

Meerding, Netherlands [36]	EQ-5D	2904	2.5 months	0.86	all injury, age 15+
			2.5 months	0.63	hospitalized 15+
			5 months	0.74	hospitalized 15+
			9 months	0.74	hospitalized 15+

Polinder, Netherlands [3]	EQ-5D	525	2.5 months	0.92	all injury, age 4-15
		379	5 months	0.96	all injury, age 4-15

		366	9 months	0.98	all injury, age 4-15

Polinder. Netherlands [37]	EQ-5D	3231	2.5 months	0.60	all injury, age 15+
			5 months	0.70	all injury, age 15+
			9 months	0.76	all injury, age 15+

			24 months	0.73	all injury, age 15+

Sutherland, UK [32]	SF-6D	200	2 months	0.61	Admitted, age 16-70
			6 months	0.62	Admitted, age 16-70

Watson, Australia [34]	SF-6D	186	pre-injury	0.91	Admitted, age 18-74

		186	12 months	0.73	Admitted, age 18-74

**SEVERE INJURY PATIENTS**					

Gabbe, Australia [10]	HAQ	243	discharge	0.44	ISS > 15, age 15-80
			6 months	0.54	ISS > 15, age 15-80

Holbrook US [12-14]	QWB	1048	discharge	0.40	GCS > 11, age 18+
			6 months	0.63	GCS > 11, age 18+
			12 months	0.67	GCS > 11, age 18+

			18 months	0.68	GCS > 11, age 18+

Holtslag, Netherlands [64]	EQ-5D	335	15 months	0.69	ISS > 15, age 15+

Korosec, Slovenia [61]	EQ-5D	98	24 months	0.72	ICU patients

Soberg, Norway [11]	WHODAS II *	105	6 weeks	0.59	ISS > 15, age 18-67
			12 months	0.72	ISS > 15, age 18-67

			24 months	0.73	ISS > 15, age 18-67

* WHODAS II disability weights were reversed to utility scores

**QOL **= Satisfaction with Quality of Life instrument; **EQ-5 D **= European Quality of Life instrument -5 dimensions; **SF-6D **= Medical Outcome Study Short Form-6 dimensions; **HAQ **= Health Assessment Questionnaire; **QWB **= Quality of Well Being Scale; **WHODASII **= World Health Organization Disability Assessment Schedule version II

## Discussion

This systematic review aimed to provide greater insight into the measurement of functional outcome and recovery patterns of general injury populations in studies using a generic health state measure. There was considerable methodological variation between studies, including different settings, mixture of participants, instruments, and follow-up periods and timings of assessment. Among available generic instruments, the SF-36, FIM, GOS and EQ-5D have been most frequently used. Studies of functional outcome of the general injury population are still uncommon and generally not comparable, preventing an in-depth understanding of the HRQL experiences of injured persons. Evidence from our review lends support to the need for guidelines for the conduct of follow-up studies measuring injury-related disability.

Longitudinal studies with multiple time points measuring outcomes, and incorporating a retrospective assessment of the pre-injury situation are needed to produce valid estimates of injury-related morbidity and disability. Studies with this design provide insight into the course of recovery over time and quantify the longer-term functional consequences of injuries. There is still a lack of consensus on preferred HRQL instruments and study designs given the wide variety of different approaches that are used by the articles included in this review. This variability prevents meta-analyses necessary to refine quantification of the impact of injury on population health over time.

Almost no papers provided a description of the evaluation of the instruments used against widely accepted criteria with in the field: data quality, reliability, validity and responsiveness (e.g. the COSMIN checklist [44]). Preferably, instruments should only be widely applied within the injury field if there is acceptable evidence for these measurement criteria in the population of interest. Empirical head-to-head comparisons of different HRQL measures are needed to obtain more insight into the strengths and limitations of the multi-attribute utility measures (MAUI) to estimate utility losses in injury populations. Such head-to-head comparisons have so far been lacking. Several of the authors have recently published a paper comparing the Health Utility Index (HUI) mark 2 and 3 and the EQ-5D in 'all injury patients' [45]. However, to maximise the utility of the available sparse data there is a need for studies which develop translational or bridging metrics for the different instruments which would then allow data to be combined in meta-analyses.

The EuroSafe Group has developed guidelines which advise the use of a combination of the EQ-5D and the HUI, with assessments at 1, 2, 4 and 12 months after injury [1]. Few studies, including those published after 2007, satisfy the guidelines. Only five studies used the EQ-5D, and none was found that used the HUI. The guidance recommended the use of two instruments to measure functional outcome, but half of the studies only used one instrument. With the exception of the twelve month measurement few recommended time points were assessed. However, most of the published studies were designed before publication of the guidelines in 2007.

Different HRQL instruments assess different dimensions of health, which make comparisons of study outcomes difficult. Polinder et al. showed that the HUI and EQ-5D resulted in significantly different utilities for similar health states for a general injury population [45]. These differences have the undesirable effect that the distinct instruments yield different utilities for similar health states. Clinicians and researchers should be aware of these differences between the HRQL instruments. It is remarkable that in the 41 papers reviewed 24 different HRQL instruments were used. The use of so many different HRQL instruments might indicate that none of the instruments seems to incorporate all the attributes that one would like to, or that there is uncertainty about which instrument is best to use. Decisions regarding which HRQL measure to use will be influenced by a range of factors. For example, researchers may choose to include measures where normative population values are available or where the HRQL instrument is available in their national language. Our review shows that the choice of an instrument is also country specific (e.g. in the Netherlands the EQ-5D is very often used and in the US the SF-36). Other factors such as user fees and instrument length will also be influential. Researchers may also choose instruments based on considerations that are specific to their study which may make generalizability difficult. For example, they may choose an injury specific instrument with greater responsiveness to change for their particular study question rather than a generic instrument.

The importance of using the same generic health instruments in multiple studies needs to be raised across the injury research community. In our view, the ideal measure to quantify the burden of injuries should include all dimensions relevant to the burden of injury, produce a 0-1 range, a utility or summary score, be responsive to changes over time and not be injury specific to enable comparisons with other diseases. We think that the HRQL instruments proposed by the EuroSafe group (EQ-5D and HUI) include the majority of the relevant dimensions for measurement of the burden of injury and the instruments are suitable for 'all injury' populations and all but the youngest age ranges [45,46].

The Eurosafe recommendations were based on an assessment of whether all relevant health domains for injury patients are included, when measuring the functional consequences of injury. As a first criterion, all body functions, activities and participation domains of the International Classification of Functioning (ICF) were defined, that are relevant for a substantial proportion of injury patients: cognition, emotion, pain, problem solving, ambulation, use of hand/arm/fingers, self care, household activities, interpersonal interactions (including sexual activities), school and/or work, and recreation. Actually, none of the generic measures studied cover all the relevant domains. However, a combination of a measure focusing on the functional capacities of the patient on the one hand (such as the HUI) and a measure including social participation on the other hand (such as EQ-5D) provides the best compromise. To assess functional capacities, Functional Capacity Index (FCI [47]), the only available injury specific instrument could in principle be used, but validation studies of this measure have been few and inconclusive [19,48]. For this reason, the few studies using the FCI conducted so far, were not included in our review.

Until improved evidence based recommendations become available, the EuroSafe guidance should be adopted across the injury field to facilitate comparisons between studies and to provide greater insight into functional outcome and recovery patterns after injury. Of course, depending on the type of injuries included in future studies researchers may continue to use different assessment periods and variability in follow-up. However, if researchers can adhere to the guidelines as closely as possible, the opportunity for the improved understanding of injury outcomes will be enhanced.

It is clear that given the current state of knowledge it is difficult to summarize the functional outcome of injuries amongst the general injury population, due to wide variety of study designs, instruments used, and timing of outcome assessments. Nevertheless, this review has provided an improved insight into functional outcomes and recovery patterns of injury patients. A high prevalence of health problems during and after the first year of injury was a common finding of the studies. Among 'all injury' groups recovery occurred predominately during the first year following injury, whereas some 'more severely' injured patients also recovered by a smaller amount during the second year. Two years post-injury both groups, on average, still showed large deficits from full recovery whether measured by population norms or differences from pre-injury health status.

Several authors have recently called for further comprehensive population-level research exploring outcomes after injury, particularly for the non selective 'all injury' group [6,7]. Our systematic review is one of the few studies that has considered the measurement of HRQL in general injury populations. There is scarcity of well-standardized data on functional outcome after injury. In 2000, Krug et. al. concluded that very few population-based data on non-fatal injury outcome were available [49]. In 2002, Garratt et all [50] concluded that there was relatively little development and evaluation of quality-of-life measures for trauma patients, compared to the almost exponential growth in other fields of health (care). Also, Segui-Gomez et al. reported on the limited application of quality-of-life measures in publications in the injury field [4]. The results of our review confirm these earlier conclusions. The earlier narrative literature search of the EuroSafe Group (2007) [1] identified only 17 studies. Four of the reviewed articles were not included in our review, because they were too injury specific (e.g. vertebral fractures [51]), or because they were cross-sectional in design and unsuited for measurement of outcomes [52-54]. Our review included 28 new studies which assessed injury-related recovery or disability. Furthermore, a systematic review of HRQL following major trauma among children reported similar findings with a large variety of HRQL measures used (n = 14) [55].

With regards to future research in the injury field there is clearly a need for further empirical studies of injury outcomes which follow the general guidelines from the 2007 EuroSafe report [1]. Such studies should include a measure of pre-injury HRQL (retrospectively) using instruments that produces utility scores. Decisions regarding which HRQL measure to use is often influenced by a range of factors. Therefore, whilst researchers may use their own instruments, but should include the best validated generic instruments to ensure comparability of results across studies. The EQ5 D, combined with the HUI, is particularly recommended as an appropriate measure in 'all injury' studies due to its suitability, ease of use and also being free to use. When used, both utility scores and standard deviations should be reported. Longitudinal studies with multiple measurement points to study recovery patterns of injury patients should be a priority issue as many existing studies have had only one follow-up assessment. Since 'major trauma' patients often show further improvements after 12 months, studies focusing on such injuries should measure outcomes up to two years after injury.

Assessment of the impact of injury requires comparison with pre-injury HRQL or in the absence of such information age and gender specific population norms. Whilst population summary scores are often used new evidence suggests that such scores are significantly lower than the pre-injury summary scores of injury patients [27]. This implies that using population scores as a baseline results in an underestimation of the impact of the injury. Pre-injury scores were collected in the UK Burden of Injury Study and an Australian study though the validity of these 'pre'-injury data has been questioned as they are collected after the injury and may be prone to recall bias [27,33,41]. Comparison with population norms is also prone to bias as injured people are unlikely to be a random sample of the general population and adjustment may not be possible for unmeasured confounders.

## Conclusions

In conclusion, this review shows that there is considerable variation in study design between studies measuring HRQL of general injury populations. It is also clear that recently developed guidelines are not yet being followed. Adherence to such guidelines would facilitate comparability across studies which would produce improved estimates of injury disability and recovery patterns. There is also a need for the development of bridging tables which would allow direct comparison of the results of studies using different instruments. Such tables would be a helpful step in supporting formal meta-analyses of the results of studies using different instruments.

There are still major gaps in our understanding of the impact of injury on personal and population health. Consistently collected empirical data across countries would support the production of more valid burden of injury calculations, cost-effectiveness analyses of injury prevention programs and trauma care, and support continuous quality improvement of care.

## Competing interests

The authors declare that they have no competing interests.

## Authors' contributions

SP had full access to all of the data in the study and takes responsibility for the integrity of the data and the accuracy of the data analysis. *Review (literature search and data extraction): *SP and EB. *Study concept and design: *SP, JH, EFB, VE, JL and RL. *Analysis and interpretation of data: *SP, JH, EFB, EB. *Drafting of the manuscript: *SP, EFB, JH and RL. *Critical revision for important intellectual content and approval of the manuscript: *SP, EFB, JH, RL, JL, VE.

*Study supervision: *EFB.

## Pre-publication history

The pre-publication history for this paper can be accessed here:

http://www.biomedcentral.com/1471-2458/10/783/prepub

## Supplementary Material

Additional file 1 **Search strategy PubMed**. Click here for file

Additional file 2 **Flow diagram of the reviewing process**. Click here for file

## References

[B1] Van Beeck EF Larsen CF Lyons RA Meerding WJ Mulder S Essink-Bot ML Guidelines for the conduction of follow-up studies measuring injury-related disability J Trauma 2007 62 2 534 550 10.1097/TA.0b013e31802e70c7 17297349

[B2] Haagsma JA van Beeck EF Polinder S Hoeymans N Mulder S Bonsel GJ Novel empirical disability weights to assess the burden of non-fatal injury Inj Prev 2008 14 1 5 10 10.1136/ip.2007.017178 18245308

[B3] World Health Organization International Classification of Functioning, Disabilities, and Health (ICF) 2001 Geneva: World Health Organization

[B4] Segui-Gomez M Mackenzie EJ Measuring the Public Health impact of injuries Epidemiol Rev 2003 25 3 19 10.1093/epirev/mxg007 12923986

[B5] Murray CJ Lopez AD Global mortality, disability, and the contribution of risk factors: Global Burden of Disease Study The Lancet 1997 349 9063 1436 1442 10.1016/S0140-6736(96)07495-8 9164317

[B6] Derrett S Black J Herbison GP Outcome after injury-a systematic literature search of studies using the EQ-5D J Trauma 2009 67 4 883 890 10.1097/TA.0b013e3181ae6409 19820601

[B7] Lyons RA Measuring the burden of injury Inj Prev 2008 14 1 3 4 10.1136/ip.2007.018192 18245307

[B8] Haagsma JA Belt E Polinder S Lyons RA Macey S Atkinson M Lund J van Beeck EF WP5 Injury disability indicators 2009 Rotterdam: Department of Public Health, Erasmus Medical Center

[B9] Lyons RA Polinder S Larsen CF Mulder S Meerding WJ Toet H Van Beeck E Methodological issues in comparing injury incidence across countries Int J Inj Contr Saf Promot 2006 13 2 63 70 10.1080/17457300500258682 16707341

[B10] World Health Organization Disability Assessment Schedule II http://www.who.int/icidh/whodas/

[B11] Aitken ME Tilford JM Barrett KW Parker JG Simpson P Landgraf J Robbins JM Health status of children after admission for injury Pediatrics 2002 110 2 Pt 1 337 342 10.1542/peds.110.2.337 12165587

[B12] Evanoff B Abedin S Grayson D Dale AM Wolf L Bohr P Is disability underreported following work injury? J Occup Rehabil 2002 12 3 139 150 10.1023/A:1016838510682 12228945

[B13] Gillen M Jewell SA Faucett JA Yelin E Functional limitations and well-being in injured municipal workers: a longitudinal study J Occup Rehabil 2004 14 2 89 105 10.1023/B:JOOR.0000018326.23090.63 15074362

[B14] Holbrook TL Anderson JP Sieber WJ Browner D Hoyt B Outcome after major trauma: discharge and 6-month follow-up results from the Trauma Recovery Project J Trauma 1998 45 2 315 323 10.1097/00005373-199808000-00018 9715189

[B15] Holbrook TL Anderson JP Sieber WJ Browner D Hoyt DB Outcome after major trauma: 12-month and 18-month follow-up results from the Trauma Recovery Project J Trauma 1999 46 5 765 771 10.1097/00005373-199905000-00003 10338392

[B16] Holbrook TL Hoyt DB The impact of major trauma: quality-of-life outcomes are worse in women than in men, independent of mechanism and injury severity J Trauma 2004 56 2 284 290 10.1097/01.TA.0000109758.75406.F8 14960969

[B17] Keyes KB Wickizer TM Franklin G Two-year health and employment outcomes among injured workers enrolled in the Washington State Managed Care Pilot Project Am J Ind Med 2001 40 6 619 626 10.1002/ajim.10001 11757038

[B18] Kiely JM Brasel KJ Weidner KL Guse CE Weigelt JA Predicting quality of life six months after traumatic injury J Trauma 2006 61 4 791 798 10.1097/01.ta.0000239360.29852.1d 17033542

[B19] Barell V Aharonson-Daniel L Fingerhut LA Mackenzie EJ Ziv A Boyko V Abargel A Avitzour M Heruti R An introduction to the Barell body region by nature of injury diagnosis matrix Inj Prev 2002 8 2 91 96 10.1136/ip.8.2.91 12120842 PMC1730858

[B20] Michaels AJ Michaels CE Smith JS Moon CH Peterson C Long WB Outcome from injury: general health, work status, and satisfaction 12 months after trauma J Trauma 2000 48 5 841 848; discussion 848-850 10.1097/00005373-200005000-00007 10823527

[B21] Winthrop AL Brasel KJ Stahovic L Paulson J Schneeberger B Kuhn EM Quality of life and functional outcome after pediatric trauma J Trauma 2005 58 3 468 473 discussion 473-464 10.1097/01.TA.0000153940.23471.B7 15761338

[B22] Aitken ME Jaffe KM DiScala C Rivara FP Functional outcome in children with multiple trauma without significant head injury Archives of physical medicine and rehabilitation 1999 80 8 889 895 10.1016/S0003-9993(99)90079-5 10453764

[B23] Airey CM Chell SM Rigby AS Tennant A Connelly JB The epidemiology of disability and occupation handicap resulting from major traumatic injury Disability and rehabilitation 2001 23 12 509 515 10.1080/09638280010010697 11432647

[B24] Baldry Currens JA Evaluation of disability and handicap following injury Injury 2000 31 2 99 106 10.1016/S0020-1383(99)00246-6 10748812

[B25] Baldry Currens JA Coats TJ The timing of disability measurements following injury Injury 2000 31 2 93 98 10.1016/S0020-1383(99)00244-2 10748811

[B26] Braithwaite IJ Boot DA Patterson M Robinson A Disability after severe injury: five year follow up of a large cohort Injury 1998 29 1 55 59 10.1016/S0020-1383(97)00164-2 9659483

[B27] Lyons RA Towner EE Kendrick D Christie N Brophy S Phillips CJ Coupland C Carter R Groom L Sleney J The UK burden of injury study - a protocol. [National Research Register number: M0044160889] BMC Public Health 2007 7 317 10.1186/1471-2458-7-317 17996057 PMC2225415

[B28] Hetherington H Earlam RJ Kirk CJ The disability status of injured patients measured by the functional independence measure (FIM) and their use of rehabilitation services Injury 1995 26 2 97 101 10.1016/0020-1383(95)92185-D 7721476

[B29] Sutherland Aea Recovery after musculoskeletal trauma in men and women The Journal of Trauma 2005 59 2 213 216 16096566 10.1097/01.ta.0000162730.86809.61

[B30] Lamers LM McDonnell J Stalmeier PF Krabbe PF Busschbach JJ The Dutch tariff: results and arguments for an effective design for national EQ-5 D valuation studies Health Econ 2006 15 10 1121 1132 10.1002/hec.1124 16786549

[B31] Gabbe BJ Cameron PA Williamson OD Edwards ER Graves SE Richardson MD The relationship between compensable status and long-term patient outcomes following orthopaedic trauma Med J Aust 2007 187 1 14 17 17605697 10.5694/j.1326-5377.2007.tb01108.x

[B32] Gabbe BJ Simpson PM Sutherland AM Williamson OD Judson R Kossmann T Cameron PA Functional measures at discharge: are they useful predictors of longer term outcomes for trauma registries? Ann Surg 2008 247 5 854 859 10.1097/SLA.0b013e3181656d1e 18438124

[B33] Watson WL Ozanne-Smith J Richardson J Retrospective baseline measurement of self-reported health status and health-related quality of life versus population norms in the evaluation of post-injury losses Inj Prev 2007 13 1 45 50 10.1136/ip.2005.010157 17296689 PMC2610562

[B34] Holtslag HR Post MW Lindeman E Van der Werken C Long-term functional health status of severely injured patients Injury 2007 38 3 280 289 10.1016/j.injury.2006.10.026 17250834

[B35] Meerding WJ Looman CW Essink-Bot ML Toet H Mulder S van Beeck EF Distribution and determinants of health and work status in a comprehensive population of injury patients J Trauma 2004 56 1 150 161 10.1097/01.TA.0000062969.65847.8B 14749582

[B36] Polinder S Meerding WJ van Baar ME Toet H Mulder S van Beeck EF Cost estimation of injury-related hospital admissions in 10 European countries J Trauma 2005 59 6 1283 1290 discussion 1290-1281 10.1097/01.ta.0000195998.11304.5b 16394898

[B37] Polinder S van Beeck EF Essink-Bot ML Toet H Looman CW Mulder S Meerding WJ Functional outcome at 2.5, 5, 9, and 24 months after injury in the Netherlands J Trauma 2007 62 1 133 141 10.1097/TA.0b013e31802b71c9 17215744

[B38] Janssens L Gorter WJ Ketelaar M Kramer WLM Holtslag HR Long-term health condition in major pediatric trauma: a pilot study J Pediatric Surgery 2009 44 1591 1600 10.1016/j.jpedsurg.2009.02.054 19635311

[B39] Macpherson AK Rothman L McKeag AM Howard A Mechanism of injury affects 6-month functional outcome in children hospitalized because of severe injuries J Trauma 2003 55 3 454 458 10.1097/01.TA.0000042158.79688.51 14501886

[B40] Aitken ME Tilford JM Barrett KW Parker JG Simpson P Landgraf J Robbins JM Health status of children after admission for injury Pediatrics 2002 110 337 342 10.1542/peds.110.2.337 12165587

[B41] Gabbe BJ Cameron PA Graves SE Williamson OD Edwards ER Preinjury status: are orthopaedic trauma patients different than the general population? J Orthop Trauma 2007 21 4 223 228 10.1097/BOT.0b013e31803eb13c 17414548

[B42] Polinder S van Beeck EF Essink-Bot ML Toet H Looman CW Mulder S Meerding WJ Functional outcome at 2.5, 5, 9, and 24 months after injury in the Netherlands J Trauma 2007 62 133 141 10.1097/TA.0b013e31802b71c9 17215744

[B43] Soberg HL Bautz-Holter E Roise O Finset A Long-term multidimensional functional consequences of severe multiple injuries two years after trauma: a prospective longitudinal cohort study J Trauma 2007 62 2 461 470 10.1097/01.ta.0000222916.30253.ea 17297337

[B44] Mokkink LB Terwee CB Knol DL Stratford PW Alonso J Patrick DL Bouter LM de Vet HCW The COSMIN checklist for evaluating the methodological quality of studies on measurement properties: A clarification of its content BMC Medical Research Methodology 2010 10 8 10.1186/1471-2288-10-22 PMC2848183 20298572

[B45] Haagsma JA Polinder S Havelaar AH Analysis and documentation of the results of the reveiw of existing burden of disease methods 2010 Rotterdam: Department of Public Health, Erasmus Medical Center

[B46] Polinder S Haagsma JA Bonsel G Essink-Bot ML Toet H van Beeck EF The measurement of long-term health-related quality of life after injury: comparison of EQ-5 D and the health utilities index Inj Prev 16 3 147 153 10.1136/ip.2009.022418 20570982

[B47] MacKenzie EJ Damiano A Miller T Luchter S The development of the Functional Capacity Index J Trauma 1996 41 5 799 807 10.1097/00005373-199611000-00006 8913207

[B48] Schluter PJ Neale R Scott D Luchter S McClure RJ Validating the functional capacity index: a comparison of predicted versus observed total body scores J Trauma 2005 58 2 259 263 10.1097/01.TA.0000154283.88208.9C 15706185

[B49] Krug EG Sharma GK Lozano R The global burden of injuries Am J Public Health 2000 90 4 523 526 10.2105/AJPH.90.4.523 10754963 PMC1446200

[B50] Garratt A Schmidt L Mackintosh A Fitzpatrick R Quality of life measurement: bibliographic study of patient assessed health outcome measures BMJ 2002 324 1417 1421 10.1136/bmj.324.7351.1417 12065262 PMC115850

[B51] Badia X Diez-Perez A Alvarez-Sanz C Diaz-Lopez B Diaz-Curiel M Guillen F Gonzalez-Macias J Spanish GSG Measuring quality of life in women with vertebral fractures due to osteoporosis: a comparison of the OQLQ and QUALEFFO Qual Life Res 2001 10 4 307 317 10.1023/A:1012200508847 11763244

[B52] Inaba K Goecke M Sharkey P Brenneman F Long-term outcomes after injury in the elderly J Trauma 2003 54 3 486 491 10.1097/01.TA.0000051588.05542.D6 12634527

[B53] Vles WJ Steyerberg EW Essink-Bot ML van Beeck EF Meeuwis JD Leenen LP Prevalence and determinants of disabilities and return to work after major trauma J Trauma 2005 58 1 126 135 10.1097/01.TA.0000112342.40296.1F 15674163

[B54] Zelle B Stalp M Weihs C Müller F Reiter F Krettek C Pape H Validation of the Hannover Score for Polytrauma Outcome (HASPOC) in a sample of 170 polytrauma patients and a comparison with the 12-Item Short-Form Health Survey [in German] Chirurg 2003 74 361 369 10.1007/s00104-003-0621-y 12719878

[B55] Janssens L Gorter JW Ketelaar M Kramer WL Holtslag HR Health-related quality-of-life measures for long-term follow-up in children after major trauma Qual Life Res 2008 17 5 701 713 10.1007/s11136-008-9339-0 18437531 PMC2440951

[B56] Vazquez Mata G Rivera Fernandez R Perez Aragon A Gonzalez Carmona A Fernandez Mondejar E Navarrete Navarro P Analysis of quality of life in polytraumatized patients two years after discharge from an intensive care unit J Trauma 1996 41 2 326 332 10.1097/00005373-199608000-00022 8760545

[B57] Gofin R Adler B A seven item scale for the assessment of disabilities after child and adolescent injuries Inj Prev 1997 3 2 120 123 10.1136/ip.3.2.120 9213158 PMC1067793

[B58] Gofin R Hass T Adler B The development of disability scales for childhood and adolescent injuries J Clin Epidemiol 1995 48 7 977 984 10.1016/0895-4356(94)00198-Y 7782806

[B59] Brenneman FD Redelmeier DA Boulanger BR McLellan BA Culhane JP Long-term outcomes in blunt trauma: who goes back to work? J Trauma 1997 42 5 778 781 10.1097/00005373-199705000-00004 9191655

[B60] Kopjar B The SF-36 health survey: a valid measure of changes in health status after injury Inj Prev 1996 2 2 135 139 10.1136/ip.2.2.135 9346078 PMC1067678

[B61] Korosec Jagodic H Jagodic K Podbregar M Long-term outcome and quality of life of patients treated in surgical intensive care: a comparison between sepsis and trauma Crit Care 2006 10 5 R134. 10.1186/cc5047 16978417 PMC1751058

[B62] Alves ALA Salim FM Martinez EZ Passos ADC de Carlo MMRP Scarpelini S Quality of life in trauma victims six months after hospital discharge Rev Saude Publica 2009 43 1 6 10.1590/s0034-89102009000100020 19169588

[B63] Pirente N Gregor A Bouillon B Neugebauer E Quality of life of severely injured patients 1 year after trauma. A matched-pair study compared with a healthy control group [in German] Unfallchirurg 2001 104 57 63 10.1007/s001130050688 11381763

[B64] Holtslag HR van Beeck EF Lindeman E Leenen LP Determinants of long-term functional consequences after major trauma J Trauma 2007 62 919 927 10.1097/01.ta.0000224124.47646.62 17426549

[B65] Dimopoulou I Anthi A Mastora Z Theodorakopoulou M Konstandinidis A Evangelou E Mandragos K Roussos C Health-related quality of life and disability in survivors of multiple trauma one year after intensive care unit discharge Am J Phys Med Rehabil 2004 83 3 171 176 10.1097/01.PHM.0000107497.77487.C1 15043350

[B66] Stalp M Koch C Regel G Krettek C Pape HC Development of a standardized instrument for quantitative and reproducible rehabilitation data assessment after polytrauma (HASPOC) (in German) Chirurg 2001 72 312 318 10.1007/s001040051312 11317454

[B67] Stalp M Koch C Ruchholtz S Regel G Panzica M Krettek C Pape HC Standardized outcome evaluation after blunt multiple injuries by scoring systems: a clinical follow-up investigation 2 years after injury J Trauma 2002 52 6 1160 1168 10.1097/00005373-200206000-00023 12045647

