# Supplementary material for: A systematic review of studies measuring health-related quality of life of general injury populations
Source: BMC Public Health. 2010 Dec 23;10:783. doi: 10.1186/1471-2458-10-783 (PMC3019196; doi:10.1186/1471-2458-10-783)
Supplement: Additional file 1 — Search strategy PubMed. [file 1471-2458-10-783-S1.DOC]

**Additional files**

**Additional file 1**

**Title:** Search strategy PubMed

**Description:** Searches of eligible studies were conducted in PubMed (Medline), Web of Science, Embase, and PsychInfo. Electronic search strategies were developed.

The PubMed search strategy: “(wounds and injuries OR injury OR injuries OR injured) AND ("health status indicator" OR "health status indicators" OR "health status indicators"[mesh] OR "disability evaluation" OR "disability evaluation"[mesh] OR "health status evaluation" OR "health status measure" OR SF-36 OR SF-12 OR euroqol OR eq-5d OR "health utilities index" OR hui-2 OR hui2 OR hui-3 OR hui3 OR "quality of well being scale" OR "quality of wellbeing scale" OR QWB OR "nottingham health profile" OR "sickness impact profile" OR "sickness impact profile"[mesh] OR "functional independence measure" OR "disability assessment schedule" OR WHODAS-II OR WHODAS-2 OR who-das-ii OR who-das-2) AND (statistics-and-numerical-data[subheading] OR Cohort-Studies[mesh] OR vital-statistics[mesh]) AND (eng[la] OR ger[la]) AND 1995:3000[dp]”.
