# Supplementary material for: A systematic review of studies measuring health-related quality of life of general injury populations
Source: BMC Public Health. 2010 Dec 23;10:783. doi: 10.1186/1471-2458-10-783 (PMC3019196; doi:10.1186/1471-2458-10-783)
Supplement: Additional file 2 — Flow diagram of the reviewing process. [file 1471-2458-10-783-S2.DOC]

**Additional file 2**

**Title:** Flow Diagram of the reviewing process

**Description:** Inclusion and exclusion of studies through the review process

**Screening**

**Included**

**Eligibility**

**Identification**

Records identified through database searching
(n = 6258 )

Additional records identified by screening reference lists
(n = 87 )

Records after duplicates removed
(n = 6291 )

Titles/abstracts screened
(n = 6291 )

Studies excluded
- based on title: n = 6031

-based on abstract: n= 165

Full-text articles assessed for eligibility
(n = 95 )

Full-text articles excluded
(n = 54 )

Studies included in qualitative synthesis
(n = 41 )
